# “The perfect storm”: community worker perspectives on the impact of COVID-19 on New York City immigrants and migrant-serving organizations

**DOI:** 10.3389/fpubh.2024.1387182

**Published:** 2024-05-07

**Authors:** L. Ansley Hobbs, Dima Masoud, Kathleen Cravero, Elisabeth Manipoud Figueroa, Diana Romero

**Affiliations:** ^1^Center for Immigrant, Refugee, and Global Health, CUNY Graduate School of Public Health and Health Policy, New York, NY, United States; ^2^Department of Health Policy and Management, CUNY Graduate School of Public Health and Health Policy, New York, NY, United States; ^3^Department of Community Health and Social Sciences, CUNY Graduate School of Public Health and Health Policy, New York, NY, United States

**Keywords:** organizations, immigrants, COVID-19, NYC, service support, coordination

## Abstract

**Background:**

Immigrants in New York City (NYC) have higher COVID-19 mortality than the general population. While migrant-serving organizations (MSOs) provide access to a breadth of services, they are disproportionately impacted by the COVID-19 pandemic due to staffing limitations, funding cuts, and resource limitations of communities served.

**Methods:**

Six focus-group discussions were conducted to explore the experiences of MSOs in NYC during the COVID-19 pandemic from November 2021 to March 2022. Study participants csomprised a subsample of survey respondents from a larger study identified via lists of MSOs.

**Results:**

Twenty-seven organizational representatives from 11 MSOs across NYC participated in the discussions. In addition to providing information on communities served, services offered, and organizational characteristics, the following themes emerged from the convenings: mental health challenges and resources needed for immigrants; immigration-related challenges; factors exacerbating hardships for immigrants during COVID-19; interorganizational collaborations and partnerships; policy change; and needs/requests of MSOs. MSOs provide a wide range of services as non-profit organizations and use interorganizational collaboration to improve service delivery. The proximity of MSOs to immigrant communities helps providers understand the needs of immigrants relating to the COVID-19 pandemic and factors that shape telehealth services.

**Conclusion:**

MSOs are important providers and advocates for immigration policy in the US given their relationship with the populations they serve. These findings have implications for how to support MSOs that serve immigrants in NYC. Strategies to achieve this include timelier availability and exchange of information, policies, and research as well as strengthening the experience-based advocacy of these groups.

## 1 Introduction

In March 2020, COVID-19 cases in the New York City (NYC) metropolitan area accounted for 50% of all cases in the United States (US) (1). COVID-19 had a visible impact on immigrant New Yorkers, with studies of large urban areas revealing that immigrant communities suffered higher COVID-19 mortality compared with US citizens (2). The COVID-19 positivity rate was largely concentrated in the boroughs of Queens and the Bronx, the most racially and ethnically diverse boroughs in NYC (3). The disproportionate impact of COVID-19 on immigrant communities highlights disparities in access to health care and the challenges of organizations that serve them.

Community-based organizations have been significantly affected as a result of the COVID-19 pandemic as both staff and community members face ongoing challenges related to food and housing insecurity, reduced access to social services, critical shortages in staffing, and impacted ability to provide regular programming (4). These organizations have experienced a decrease in funding since March 2020, forcing the closure of facilities in underserved communities and shifting to virtual spaces, furthering the “digital divide” between organizations and community members who cannot access services without an internet connection and/or technology devices (5). Migrant-serving organizations (MSO) are unique community-based organizations due to their cultural focus on the diverse communities they serve. These organizations may be disproportionately impacted by the COVID-19 pandemic as an individual’s immigration status affects their ability to access medical and social services, creating a government service gap which MSOs must fill. For example, asylum-seeking individuals were ineligible to apply for emergency assistance and healthcare benefits that the general population relied on during the pandemic (6). Organizations also reported reduced enrollment in health insurance for eligible immigrants due to lingering distrust in the US healthcare system because of punitive immigration policies like the Public Charge Final Rule, rescinded in March 2021 (7, 8).

To improve the ability of public health practitioners to respond to the impact of COVID-19 on MSOs and immigrant communities, we conducted focus group discussions with staff from several MSOs throughout NYC as the second phase of a survey study conducted from December 2020 through January 2021 (9). We sought to use grounded theory to better understand the experiences of MSOs during the COVID-19 pandemic, learn about their priorities, and receive input on the creation of a migrant health resource center.

## 2 Materials and methods

As a follow up to the initial survey study, we conducted a series of focus group discussions, or *convenings*, with organizational leaders from MSOs that participated in the survey phase (9). The qualitative data provides more in-depth information and context to augment findings from the survey. The research protocol was approved by the CUNY SPH Human Research Protection Program’s Institutional Review Board (#2020-0835-PHHP).

### 2.1 Sample

The qualitative study sample was derived from a subsample of 38 survey respondents. The sample was originally identified via lists of immigrant-serving organizations compiled from the New York Immigration Coalition and the New York State Department of Education’s Guide to Community-Based Organizations for Immigrants. We convened organizational respondents to the survey phase of the project to provide a report-back of the survey results. We then invited a subsample of organizations to participate in the convenings to further contextualize the survey results and discuss their plans for addressing migrant health needs.

### 2.2 Data collection

The focus group topic guide (Supplementary material) consisted of 10 key questions organized into three domains: 1. Community Description and Health Issues; 2. Collaborations and Utility of a Migrant Health Resource Center; and 3. MSO Priorities and Challenges. The semi-structured topic guide allowed for discussion of the most prominent health and social issues that communities confronted and how COVID-19 exacerbated these issue areas. Participants also discussed the extent to which their organizations collaborated with other providers to manage service demands during COVID-19, ways that a migrant health resource hub may have been useful during the pandemic, and organizational priorities and areas for future collaboration.

Focus groups were conducted via the Zoom meeting platform with three to four organizational representatives participating in each session. Each session had a moderator and co-moderator who together facilitated the discussion with the semi-structured topic guide. We received verbal and electronic consent for participation and to record the meeting from each participant. Each focus group lasted 1.5–2 h.

### 2.3 Analysis

Transcripts were cleaned against the audio recordings and de-identified. Members of the research team developed a preliminary codebook based on initial transcript review. Team meetings were held to review, discuss, and revise the code structure. Subsequent blinded coding by paired members of the team was carried out to ensure consistency in code application across analyses (10). We used the Dedoose (Ver. 9.0.54) platform for descriptive analysis involving application of the finalized hierarchical code structure, which then progressed to interpretative analysis wherein we identified overarching themes, including noteworthy concordant and discordant organizational experiences. Transcripts were independently coded by two members of the research team.

## 3 Results

Six focus group convenings were held between November 2021 and March 2022, comprising 27 participants from 11 MSOs in NYC. Findings were categorized in the following categories: MSO and community profiles; mental health of immigrants; immigration-related challenges; factors exacerbating hardship for immigrants during COVID-19; interorganizational collaboration and partnerships; policy change; and needs/requests of MSOs (Table 1).

**Table 1:** Results: categories/themes and sub-themes.

Categories	Themes/Sub-themes
MSOs: communities served, services offered and organization characteristics	Ethnic origin of the community served
	Neighborhoods of communities served
	Various types of services offered including legal, educational, health, and referral services
	How long they have worked in/served these communities
	How the demographics of their staff represent members of this defined community (e.g., race/ethnicity, geographic location, similar socioeconomic experiences, etc.)
Mental health experiences and resources for immigrants	Taboo to discuss/seek care
	Psychosocial impacts and trust
	Isolation
	Re-experiencing trauma
Immigration-related challenges and needs	Lack of understanding of access to care among patients w/various legal statuses (e.g., asylum-seeking patients)
	Workers that are unfamiliar with benefits and healthcare programs for patients w/ various legal statuses
	Barriers: documentation status, forms of identification, language, cultural differences, transportation, and technology
Factors exacerbating hardships for immigrants during COVID-19	Socioeconomic issues
	Service barriers and exclusions (housing insecurity, food insecurity, etc.)
Interorganizational collaborations and partnerships	Existing relationships and coalition building
	Interest in future collaboration
Policy change	Policy examples producing disparate outcomes (e.g., Public Charge, CARES Act)
	Making concrete changes to improve the health and lives of immigrants
	Recommendations for policies based on research/evidence-based findings; suggestions regarding where more evidence is required
Needs and recommendations from MSOs	The need for additional funding, language interpretation assistance, open-source resource repositories, cultural competency/humility training, interorganizational referrals for holistic service provision, and increased research opportunities
	Resources and training to manage MSO employee stress as they aim to meet the needs of the populations they serve

### 3.1 MSOs: communities served, services offered, and organization characteristics

Many participating organizations were founded and run by community members who saw an unmet need and decided “to jump into that landscape because they were a part of us, we were a part of them” (FG5). Individual participants represented NYC-based MSOs providing services in the Bronx, Manhattan, Brooklyn, Queens, Staten Island, the Hudson Valley region, and New Jersey. Communities served included immigrants, refugees, asylum seekers, women, youth, queer, and low-income individuals, and predominantly Hispanic/Latino, African, Middle Eastern, and South Asian populations. Staff demographic characteristics often reflect the communities they serve, including race/ethnicity, socioeconomic background, and place of residence. Most participants were multilingual, speaking both the language of their client population and the language of the host community. This close affiliation with community identity and experiences was said to foster greater cultural humility among MSO workers and build trust among community members.

**Figure 1:**
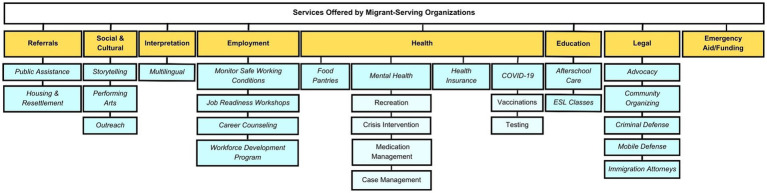
Services offered by migrant-serving organizations.

**Figure 2:**
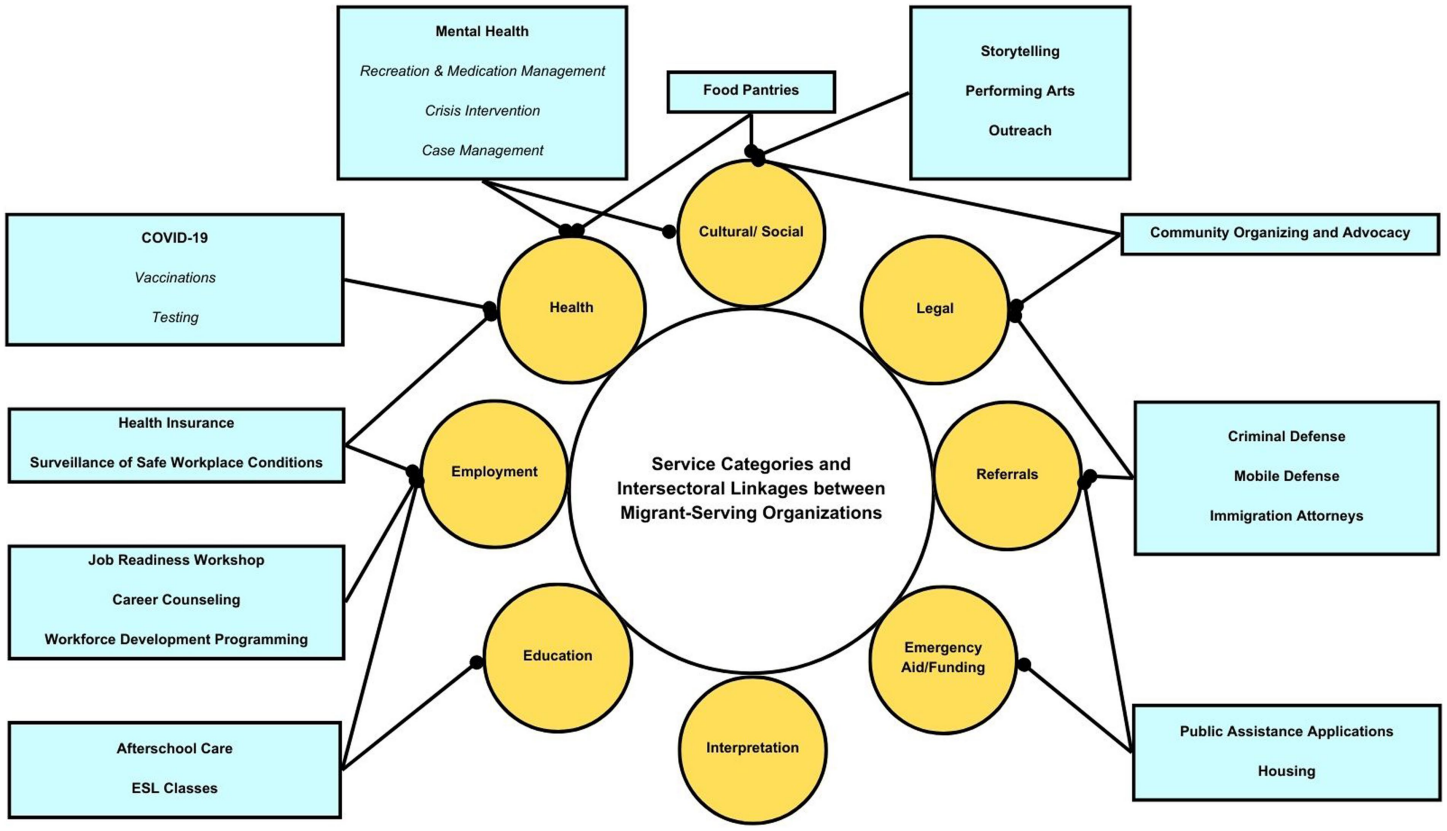
Service categories and intersectoral linkages between migrant-serving organizations.

Participating organizations varied in size and capacity, from smaller (e.g., serving 300 clients) neighborhood-specific organizations to larger organizations (e.g., serving 4,000+ clients) across NYC. They offer a wide range of services in the broad areas of health, education, legal, cultural, social, referrals, employment, and interpretation. As immigrant communities are impacted by consecutive and compounding emergencies (e.g., COVID-19, violent crime, xenophobia/racism, displacement from natural disasters/war), organizations indicated that they must be equipped to respond to the impact and trauma of parallel crises, making needed service delivery more complex. All participants reported shifting their services to adapt to social distancing protocols, while still trying to meet diverse community needs during COVID-19. Many organizations reported having served as testing and vaccination centers throughout the five boroughs. Figure 1 provides a summary of the range of services MSOs offered while Figure 2 identifies intersectoral linkages between service categories.

### 3.2 Challenges impacting mental health

Participants identified a growing need to improve access to mental health services to address the psychosocial health of newly arrived immigrants following potentially traumatic, violent, and/or life-threatening experiences in their home countries or while traveling to the US. However, several participants noted that mental health struggles and diagnoses are viewed as taboo in some immigrant communities. One participant put it this way:

“Mental health services have such a stigma in a lot of other cultures, and people just don't want to admit that they may have issues, that their children may have issues, and that they need certain services.” (FG2)

Nonetheless, some communities appeared to overcome this stigma in response to compounding stressors during COVID-19, with the demand for mental health referrals during the pandemic skyrocketing. One participant (FG4) reported a 350% increase in referrals to a mental health counseling program from 2020 to 2022. Yet, the sudden increased demand for mental health services during the pandemic exposed the lack of available clinicians in the field, particularly those who are bilingual in English and indigenous languages (e.g., Wayuu, Nahuatl, Maya, etc.).

Living in fear of exploitation, coercion, discrimination and racism, and threats of deportation were frequently reported by MSO participants to have serious mental health impacts on the immigrant communities they serve. During both migration and after resettlement, power imbalances are frequently used to coerce or exploit immigrant communities, with one participant describing how “the landlords are using immigration status against our clients and then clients just do not feel empowered to stay because it’s just so scary and uncomfortable to stay in these settings” (FG5). Threats related to immigration status were said to also increase the sense of isolation within immigrant communities in lock-down and exacerbate pre-existing mental health impacts of COVID-19, including the fear of falling ill without health insurance.

One distinct group frequently mentioned in relation to mental health were students. Living in multigenerational households with limited access to the required technology for remote learning, students had to overcome tremendous hardships during the COVID-19 pandemic. Participants described immigrant students’ mental state as “fraying” and reported an uptick in cases of anxiety, depression, absenteeism, and behavioral issues. Others flagged developmental issues among younger students who would normally be learning to socialize in a classroom environment.

### 3.3 Immigrant-specific barriers to care

Limited understanding of the city and state health care and social service systems was seen in both immigrant populations as well as with providers and service navigators. Participants detailed interactions with New York State Human Resources Administration (HRA) workers who were unfamiliar with the benefit and healthcare programs for individuals with various documentation statuses, stating that “case managers at clinics or hospitals very often have this amalgamate in their heads that if somebody is a non-citizen, they are not eligible for any benefits, which is completely not true” (FG1). This issue is especially pronounced for undocumented and asylum-seeking patients. This confusion leads to lower levels of service enrollment, thereby impacting their access to affordable health care. As a result, individuals may not receive preventative health services and seek care at hospitals only during emergency situations. One participant noted that her clients sometimes “enter the emergency rooms, and then they find themselves with a huge bill that they do not know how to pay for or to navigate, and then this scares them even more to find preventive services” (FG2).

The fear of exploitation leads to an erosion of trust between immigrants and government institutions, programs, and health systems. Mistrust “in the people who are making decisions for our lives” was frequently identified as an abstract but pervasive barrier to care and to COVID-19 vaccine uptake. While telehealth was intended to provide safe, remote access to care during the pandemic, there were challenges for immigrants with low technological literacy, access to the internet, and/or necessary devices. While some organizations offered service coordination over the phone in many languages, some individuals were wary of providing their personal information outside of an in-person setting. MSOs emphasized how community navigators, culturally competent providers, and culturally appropriate services can help to overcome these challenges.

Language and other cultural factors can serve as barriers. Participants frequently noted a lack of interpretation services and culturally sensitive personnel in healthcare settings. Culture was also an important consideration for food pantries, as some might be unable to eat certain foods due to religious practices or cultural preferences. Participants recommended that programming for immigrant communities use culturally competent design and case management.

Another immigrant-specific barrier to health and social services is a lack of identification. Many health and social service programs require specific forms of identification to access services, such as a passport, country-issued ID, driver’s license, or an NYC-issued IDNYC. Identification issues are most pronounced for undocumented immigrants who may have had their belongings confiscated in migrant detention centers.

### 3.4 Socioeconomic factors exacerbating hardships for immigrants during COVID-19

Many participants discussed the social determinants of health and the ways in which COVID-19 exacerbated existing inequities. They reported that community members frequently told them about the “economic impact,” “loss of a primary breadwinner,” and added “economic pressure” due to job loss or decreased hours. One participant described the circumstances as “the perfect storm”:

“We really saw that kind of combination of economic strain and mental health strain and insufficient access to public resources as that kind of perfect storm of circumstances that really exacerbated the mechanisms by which families are able to continue to remain well.” (FG4)

Immigrants experienced economic challenges more acutely than others due to several key factors. One participant noted the “limited access to some of the essential federal and state benefits that have been afforded that folks aren’t able to get access to, whether that’s stimulus checks, excluded workers’ funds, and child tax credits” (FG4). In addition to dealing with unequal benefits and emergency income assistance, the pandemic also slowed the pathways to acquiring a work permit. Work permit backlogs jumped from 6 months to 1 year, driving more job-seeking immigrants into the informal job sector and increasing their risk of labor exploitation and COVID-19 infection. Some organizations sought to overcome economic precarity and exclusion by creating their own emergency relief funds to finance basic needs.

With greater economic precarity came increased housing and food insecurity. Immigrants did not always qualify for relief available through eviction moratoriums because, according to one participant (FG5), “their housing arrangement was already fairly informal.” One participant (FG2) reported that community members took out loans to cover the cost of housing, causing “a lot of stress and anxiety” and further exacerbating financial hardship. Participants also noted high levels of food insecurity causing additional hardship for immigrant families, as well as pressure on community health workers to address this need. Several organizations described the ways in which they expanded their food distribution operations during COVID-19 through collaboration with other organizations and governmental stakeholders.

### 3.5 Interorganizational/sectoral collaborations and partnerships

MSOs discussed the importance of coalition-building to further the mission of their work and “serve as a bridge” to communities, working to “identify policy priorities, raise awareness, [and] garner political support” (FG4). Others noted that they benefited from engaging with a wide range of groups and service providers creating innovative and impactful community-engaged interventions. Examples they provided included expanding food distribution services, opening new testing and vaccination centers, fundraising, and distributing disaster and emergency relief funds, serving as community navigators for the NYC government to increase health care access, and hosting arts and cultural groups for mental health. However, smaller, neighborhood-specific organizations focused on their day-to-day operations struggled to find time to commit to external projects. Relatedly, larger organizations discussed how they are frequently tasked with leadership roles in coalitions but struggle with mobilizing disengaged or absent coalition members. In the context of COVID-19, participants felt that the pandemic “actually helped smooth out the referral process” by allowing government agencies and organizations to work “in a thoughtful way, that maybe we were not thinking about before” (FG5). This was in part due to the move to virtual coordination mechanisms, allowing for electronic instead of in-person office encounters.

Referral processes varied, with some organizations creating their own systems, while others used existing external systems, such as NowPow (now called Unite Us) (11). Regardless of system used, participants described possible improvements to make systems more accessible to MSOs and community members (e.g., no paywall or membership requirement). One participant (FG4) commented that existing referral systems were like searching for a “needle in a haystack” because there are so many services listed within NYC but only a fraction of those services are available in the languages required by community members. Participants recommended creating migrant-specific referral systems or revising them to include migrant-sensitive filters for language spoken, service cost (e.g., insurances accepted, sliding scale, free), and cultural competencies of providers (e.g., course certifications, consent to community terms and conditions for appropriate care).

### 3.6 Policy change

Participants emphasized the need for improvements to both state and federal immigration policy. One participant (FG2) characterized immigration law in the US as “a maddening roller coaster” even prior to the COVID-19 pandemic. The Public Charge policy was cited most frequently as exacerbating existing disparities in service access through misinformation and fear, with a one participant explaining:

“Government policies and political rhetoric, in particular the changes to the definition of Public Charge that occurred in the previous administration, created an extremely severe chilling effect for utilization of all services…. We saw that that in fact led many people to just disengage with any sort of institutions that they felt to be even tangentially related to the government.” (FG4)

Immigration proceedings were also impacted as court closures led to stalled cases, leaving both immigrants and legal aid groups wondering “whether the case was going to even be heard” (FG1) while extending their precarious circumstances. Emergency response policies offering some relief (e.g., CARES Act) were not distributed across-the-board early on, with immigration status determining if individuals qualified for assistance (12). Participants highlighted this limited access to early emergency assistance as a key example of immigrants being ineligible for much needed support. However, participants (FG4) did point to other emergency response policies that proved beneficial to migrants at the city level, including an additional emergency rental assistance program and emerging economic relief programs. At the state-level, the Excluded Workers Fund was cited as an essential resource for many families. Taken together, one participant (FG6) emphasized how their organization and community members were dealing with “the fallout of the expectations of Biden-Harris,” via advocacy at the federal and state levels.

### 3.7 Needs of and recommendations from MSOs

The historic disinvestment of the social service sector over the past decade was raised across focus groups, with participants feeling that “we are doing a lot of work that they [the government] are not doing, so, it would be nice to be paid for that” (FG1). They added that most government funding is for project-based “band-aid solutions” instead of long-term investment in the social service sector. Additionally, prioritizing project-based funding with large organizations means government funding becomes “inaccessible to […] small organizations” (FG1).

Participants also pointed to the need for resources and training to manage the stress and pressure felt by MSO staff. Specifically, they discussed how their and partnering organizations are “always overwhelmed and overstretched” (FG2), “very tired,” and struggling with the “day-to-day slog that is starting to wear and tear on us” (FG6).

Recommendations put forward fit into three categories to support capacity strengthening: language translation and interpretation assistance, open-source resource repositories, and improved referral systems. Language translation and interpretation assistance was among the most frequent requests. Organizations frequently raised the need for “competent, culturally informed translation” (FG2) and suggested that academic institutions with diverse student bodies could mobilize multilingual students to serve as interpreters and translators for MSOs.

Open-source resource repositories of publications, policy briefs, and community health materials would be most helpful if made “understandable, accessible, and culturally and linguistically competent” (FG2). Moreover, participants requested resources be vetted to include best policies and practices which “are adaptive to […] the particular challenges that immigrant community members are facing” (FG4). Instead of simply posting resources on a hub, participants discussed ways to promote engagement with new research through, for example, webinars with “a face to identify with these resources” (FG1).

The final recommendations noted how referral systems, including provider maps, could be improved by making them searchable by geographic region, service type, expertise, languages available, cost, and populations served. Participants (FG2) noted the need for an expert directory of medical and mental health professionals who can provide affidavits and forensic evaluations. Organizations felt that for it to be most effective it must adhere to clear community standards to ensure the quality of referrals—providers can opt in but must uphold community standards, “not just have case management. If it’s not culturally appropriate and sensitive, it’s not necessarily very helpful. You may hand them off to a case manager and that person may walk away from the case manager and never go back” (FG2).

## 4 Discussion

The study findings highlight the additional challenges that MSOs encountered due to the COVID-19 pandemic while articulating specific health and social needs and priorities of immigrant communities in NYC. We discuss four categories of key take-aways below.

### 4.1 MSOs provide a wide range of services

MSOs provide a wide range of services (including some that did not exist prior to COVID-19) partly because immigrants may avoid services from government entities due to their immigration status and fear of deportation (13, 14). As a result, MSO referrals and organizational collaborations were essential even when similar services were available from the city government.

### 4.2 Increased demand for mental health services during COVID-19

Upon arrival in the US, immigrants face a new set of challenges as they try to acclimate to a new country, adding additional stress (15, 16), anxiety (15, 16), and sometimes post-traumatic stress disorder (16, 17). One main barrier to accessing mental health services is the associated taboo or stigma. This is consistent with the literature, as stigma has been found to be the primary cultural barrier to mental health in immigrant communities within the United States (11–13). An important aspect that we identified was the tremendous increase in mental health referrals for immigrants during COVID-19. The move to virtual services was seen as a limitation for some of the organizations’ clients, but also as a strength for streamlining service referrals between organizations and city programming. It is essential to better understand how taboos regarding seeking mental healthcare appear to have been overcome, how the positive aspects of mental telehealth can be replicated, notwithstanding the persistent scarcity in native-language providers.

### 4.3 Immigration policy changes

Study participants emphasized the importance of scrutinizing policies and programs in terms of their potential impact on immigrant communities. As their organizations were responding to the ramifications from resurrected immigration policies like the Public Charge Final Rule (8), lawyers were arguing cases in the context of federal government policies such as the Zero Tolerance Immigration Enforcement Policy (18), the Migrant Protection Protocols (19), and the Third Country Asylum Rule (20). As our results suggest, such policies had a negative impact on the communities the MSOs serve. Policies developed in line with immigrants’ needs can mitigate fear and distrust as a result from anti-immigration policies. An example is the Excluded Workers Trust, which was widely celebrated by many immigrant communities compared to the Public Charge Rule (21).

### 4.4 The need for reliable, up-to-date, easily accessible referral, and policy information

Participants expressed a strong desire for a comprehensive source of information on available services offered by other organizations, policies and lessons learned by other groups. In response to this, the study team initiated the development of Immigrant Provider Action Center with a diverse community advisory board to guide its development (22).

## 5 Conclusion

MSOs were disproportionately impacted by COVID-19 pandemic in addition to the set of challenges they faced prior to the pandemic. As such, the study points to the importance of protecting those organizations and supporting them in a preventative manner for public health emergencies in the future. This can be translated into better funding, staff resources, and the maintenance and enhancement of resources available to serve the multicultural and multilingual immigrant population in NYC.

The large number of MSOs in NYC necessarily means that not all perspectives will have been captured in this study. Additionally, we did not explicitly focus on undocumented immigrants which may have resulted in underestimating the challenges MSOs encounter in serving this group (23). However, the almost 30 focus group participants from 11 MSOs provided rich information on the diverse experiences and needs of the migrant communities they serve, as well as recommended strategies going forward. This in-depth data provided valuable context and details augmenting survey findings from the prior phase of this project (9). The needs and recommendations that are highlighted in this study provide clear “next steps” for public health professionals and organizations to consider in partnership with MSOs to address the challenges they face in addressing the health needs of the immigrant communities they serve.

## Data availability statement

The raw data supporting the conclusions of this article will be made available by the authors, without undue reservation.

## Ethics statement

The studies involving humans were approved by CUNY SPH Human Research Protection Program’s Institutional Review Board (#2020-0835-PHHP). The studies were conducted in accordance with the local legislation and institutional requirements. The participants provided their written informed consent to participate in this study.

## Author contributions

LH: Conceptualization, Data curation, Formal analysis, Methodology, Project administration, Supervision, Visualization, Writing – original draft, Writing – review & editing. DM: Data curation, Formal analysis, Investigation, Methodology, Visualization, Writing – original draft. KC: Conceptualization, Funding acquisition, Project administration, Supervision, Writing – review & editing. EF: Funding acquisition, Project administration, Supervision, Writing – review & editing. DR: Conceptualization, Data curation, Formal analysis, Methodology, Supervision, Writing – review & editing.
