# Supplementary material for: “The perfect storm”: community worker perspectives on the impact of COVID-19 on New York City immigrants and migrant-serving organizations
Source: Front Public Health. 2024 May 7;12:1387182. doi: 10.3389/fpubh.2024.1387182 (PMC11106475; doi:10.3389/fpubh.2024.1387182)
Supplement: Supplementary file 1 [file Data_Sheet_1.docx]

# Annex

**MSO Convening/FG Topic Guide**

*Hello everyone and thank you for joining us! Our goal today is to obtain a deeper understanding of the health issues facing your communities -- beyond the survey from earlier this year -- and incorporate your input on our planned activities going forward. We provided brief summaries of your organizations in advance so we could get right into the discussion. That said, let's quickly:*

● *introduce ourselves;*

● *your organization and position there; and*

● *share three words that describe the communities you serve.*

[I. Community Description and Health Issues]

***First, we’ll start with some information about the communities your organizations work with.***

1. So considering that we shared organizational summaries in advance of this meeting, it would be great if there’s *anything you want to add* to that in describing the communities you serve and the *main health issues* that they confront on a day-to-day basis.

Probe (If only mentioned *medical* conditions): What type of *mental* health issues are of concern?

Probe: How do you currently provide resources/services to address these medical/mental health concerns?

2. Now, thinking about the *sources or causes* of these health problems, what is your sense about those that affect the communities you serve?

3. From your experiences over the past almost 2 years, what would you say have been the *main effects of COVID-19* on the communities you serve?

Probe: Were/are there barriers/factors that *exacerbated* hardships during the pandemic (e.g. fear, immigration policies, job loss, food insecurity)?

[II. Collaborations and Utility of a Migrant Health Resource Hub]

***These next questions pertain to your organizational collaborations and the idea of a “Migrant Health Resource Hub” hosted by the CIRGH at CUNY SPH.***

4. How would you describe the *extent to which* (nature of) you(r) *collaborate*(ion) with other migrant-serving organizations in NYC/S?

Probe: Do these collaborations ‘work’? Thoughts about if/how can be improved?

Probe: How about collaborations related specifically to *migrant health policy issues*?

5. In your view, what would be the most *valuable or useful services* you could receive from a Resource Hub focusing on migrant health?

[“BREAK”: present project proposal and briefly describe CIRGH’s ideas for it]

6. Given what we’ve just described as our early thoughts for a Resource Hub, what are *your impressions/thoughts/recommendations for our Center’s work* on such an initiative, keeping in mind that it is intended to be a ‘resource’ for MSOs such as yours, as well as policymakers, and researchers in the field?

Probe: *How* do you think a collaboration/partnership between your organizations and our Center could be of most service to your work with migrant communities?

[III. MSO Priorities and Challenges]

***Now we are going to switch gears and discuss priorities and challenges going forward.***

7. Thinking about next year (2022), what are the *main priorities* your organization plans to address (for the communities you serve)?

Probe: How about the main *health and social* priorities? *Policy (and advocacy)* priorities?

8. And what do you expect will be the main *challenges* for your organization during this time period?

Probe: Thoughts/plans to address them?

9. Finally, what are your thoughts re planning for *future* *health-related challenges/crises* (e.g., other pandemics, climate issues, etc)?

Probe: What would be the key issues regarding migrant populations to address *now* (e.g., in preparation)?

10. Wrap-up: Given all the issues we’ve just discussed, could you *tell us in 1 sentence* what would be the *key message/take-away* you would want to leave us with?
